# Impact of multisectoral health determinants on child mortality 1980–2010: An analysis by country baseline mortality

**DOI:** 10.1371/journal.pone.0188762

**Published:** 2017-12-06

**Authors:** Robert L. Cohen, John Murray, Susan Jack, Sharon Arscott-Mills, Vincenzo Verardi

**Affiliations:** 1 United States Agency for International Development, Washington, DC, United States of America; 2 Independent Consultant, Iowa City, Iowa, United States of America; 3 University of Otago, Dunedin, New Zealand; 4 ICF, Fairfax, Virginia, United States of America; 5 Université Libre de Bruxelles, Brussels, Belgium; National Academy of Medical Sciences, NEPAL

## Abstract

**Introduction:**

Some health determinants require relatively stronger health system capacity and socioeconomic development than others to impact child mortality. Few quantitative analyses have analyzed how the impact of health determinants varies by mortality level.

**Methods:**

149 low- and middle-income countries were stratified into high, moderate, low, and very low baseline levels of child mortality in 1990. Data for 52 health determinants were collected for these countries for 1980–2010. To quantify how changes in health determinants were associated with mortality decline, univariable and multivariable regression models were constructed. An advanced statistical technique that is new for child mortality analyses—MM-estimation with first differences and country clustering—controlled for outliers, fixed effects, and variation across decades.

**Findings:**

Some health determinants (immunizations, education) were consistently associated with child mortality reduction across all mortality levels. Others (staff availability, skilled birth attendance, fertility, water and sanitation) were associated with child mortality reduction mainly in low or very low mortality settings. The findings indicate that the impact of some health determinants on child mortality was only apparent with stronger health systems, public infrastructure and levels of socioeconomic development, whereas the impact of other determinants was apparent at all stages of development. Multisectoral progress was essential to mortality reduction at all baseline mortality levels.

**Conclusion:**

Policy-makers can use such analyses to direct investments in health and non-health sectors and to set five-year child mortality targets appropriate for their baseline mortality levels and local context.

## Introduction

Between 1990 and 2015, global under-five child mortality (U5MR) declined 48 percent [1]. Progress varied across countries, even where levels of income were similar [2]. Despite these improvements, in 2015, 6.6 million children died before age five (44% as newborns), most from preventable causes [1]. Key health determinants commonly associated with rapid mortality declines in low- and middle-income countries (LMICs) include evidence-based health interventions such as skilled attendance at birth, and immunizations, and multisectoral socioeconomic improvements such as per capita wealth, and girls’ education [3–7]. However, there are limited data from LMICs on how these determinants are associated with mortality reduction in countries at differing baseline child mortality levels.

During the nineteenth and twentieth century, countries that are now high-income reduced child mortality alongside improvements in socioeconomic development [8]. In the early stages of development, with limited access to health services and technologies, most mortality reduction was achieved through improved nutrition, access to clean water, sanitation and hygiene (WASH), education, reduced fertility and improved living standards [9–14]. As immunizations and antimicrobial interventions were introduced and coverage of interventions improved, morbidity and mortality from infectious diseases declined dramatically [15, 16]. Over time, newborn deaths assumed an increasing fraction of all child deaths [17].

These past patterns of U5MR decline observed in now high-income countries may not apply to LMICs today, where significant reductions in mortality are possible even without fast economic growth [5, 18]. In today’s LMICs, the availability of development assistance for health [19], improved interventions and information technology enable countries to make strategic choices not previously available. However, the effectiveness of some interventions may still depend on a country’s baseline health context and stage of socioeconomic development.

This paper aimed to investigate how improvements in multisectoral health determinants from 1980–2010 were associated with improvements in U5MR at different baseline mortality levels. We hypothesized that improvements in variables requiring less health systems capacity, public infrastructure and socioeconomic development, will be associated with U5MR reduction at all mortality levels, while other determinants will only be associated with mortality reduction at lower mortality levels since they require greater capacity in each of these areas for impact. Quantifying the impact of health determinants, especially how such impact varies with local context, may be useful for policy-makers designing health plans for their unique health challenges.

## Materials and methods

### Data management

#### Country selection

All 193 United Nations member states were considered eligible for the analysis. Countries were excluded using the following criteria: World Bank classification as high income in 1990, the baseline year for the Millennium Development Goals (MDGs), (GDP per capita >$7,620 in 1990 US dollars); population fewer than 100,000 (to avoid disproportionate impact on the analysis from very small countries); lacking data for U5MR (deaths before age 5 years per 1,000 live births). By these criteria, 152 LMICs were eligible for the analysis (S1 Table).

#### Mortality levels

We examined U5MR distributions in 1990 and divided countries into four baseline mortality groups: high, moderate, low, and very low mortality. These corresponded to U5MR per 1,000 live births levels of high (>180), moderate (100.1–180), low (50.1–100), and very low (<50.1).

#### Variable selection

We constructed the dataset for this analysis as described in previous papers for the Success Factors for Women’s and Children’s Health study series [20, 21]. Briefly, we assembled a dataset of key health determinants already identified in the literature to have an established relationship with child mortality reductions. We categorized these determinants into fourteen different policy areas (Table 1).

**Table 1:** Summary of 52 independent and 1 dependent variable in 14 policy areas, 1980–2010^1^.

Variable	Policy Area	N	Mean	S.D.	Min	Max
Debt forgiveness grants, per capita (constant 2005 US$)	GDP per capita	340	11.7	26.5	-4.5	165.2
Log(GDPpc PPP Constant 2011 international $)	GDP per capita	708	8.5	1.1	5.0	10.8
Log(GDPpc constant 2005 US$)	GDP per capita	924	7.4	1.2	4.0	10.0
GINI index	Poverty	368	41.8	9.9	16.4	67.4
Poverty gap at $1.25 a day (PPP) (%)	Poverty	370	8.4	12.0	0.0	66.6
Poverty gap at $2 a day (PPP) (%)	Poverty	370	15.8	17.6	0.0	77.9
Births attended by skilled health staff (% of total)	Health systems [service delivery]	478	76.1	27.2	5.0	100.0
Pregnant women receiving prenatal care (%)	Health systems [service delivery]	340	81.2	19.3	15.4	100.0
Hospital beds (per 1,000 people)	Health systems [infrastructure]	675	3.7	3.4	0.1	14.3
Nurses and Midwives (per 1,000 pop)	Health systems [human resources]	258	2.8	2.6	0.0	12.5
Physicians (per 1,000 people)	Health systems [human resources]	812	1.2	1.3	0.0	9.8
Log Health expenditure per capita, PPP (constant 2005 international $)	Health financing	580	5.2	1.2	2.2	7.9
Out-of-pocket health expenditure (% of total expenditure on health)	Health financing	580	38.8	19.0	2.6	99.7
Newborns protected against tetanus (%)	Immunizations	614	58.7	27.4	1.0	96.0
Immunization, DPT (% of children ages 12–23 months)	Immunizations	917	72.2	25.7	1.0	99.0
Immunization, measles (% of children ages 12–23 months)	Immunizations	898	72.0	24.6	1.0	99.0
Malaria incidence, per 1,000 pop	Malaria or HIV	592	31.0	67.2	0.0	546.6
Prevalence of HIV, total (% of population ages 15–49)	Malaria or HIV	540	2.3	4.7	0.1	27.5
Contraceptive prevalence (% of women ages 15–49)	Fertility	451	42.1	22.9	0.8	96.0
Fertility rate, total (births per woman)	Fertility	1057	4.1	1.9	1.1	9.2
Lag 5 years TFR	Fertility	1054	4.4	2.0	1.1	9.2
Exclusive breastfeeding (% of children under 6 months)	Nutrition	234	32.3	19.9	1.0	88.4
Malnutrition prevalence, height for age (% of children under 5)	Nutrition	350	31.8	15.6	1.4	70.9
Malnutrition prevalence, weight for age (% of children under 5)	Nutrition	355	16.9	12.6	0.5	66.8
Prevalence of undernourishment (% of population)	Nutrition	563	21.3	15.3	5.0	76.4
Prevalence of wasting (% of children under 5)	Nutrition	345	7.8	5.3	0.5	27.8
Control of Corruption: Estimate	Governance	602	-0.4	0.7	-2.1	1.5
Government Effectiveness: Estimate	Governance	602	-0.4	0.7	-2.3	1.5
Political Stability and Absence of Violence/Terrorism: Estimate	Governance	607	-0.4	0.9	-3.2	1.5
Regulatory Quality: Estimate	Governance	602	-0.3	0.8	-2.5	1.6
Rule of Law: Estimate	Governance	611	-0.4	0.8	-2.4	1.4
Voice and Accountability: Estimate	Governance	614	-0.3	0.9	-2.2	1.5
Electric power consumption (kWh per capita)	Infrastructure	687	1637.5	1755.3	13.3	9741.3
Rail lines (total route-km)	Infrastructure	479	7215.1	14251.7	259.0	87681.7
Road Density, (km/km2)	Infrastructure	535	0.5	0.9	0.0	7.9
Roads, paved (% of total roads)	Infrastructure	493	43.5	31.3	0.8	100.0
Improved sanitation facilities (% of population with access)	Water and sanitation	710	61.1	31.3	2.4	100.0
Improved sanitation facilities, rural (% of rural population with access)	Water and sanitation	713	52.8	33.4	0.0	100.0
Improved water source (% of population with access)	Water and sanitation	718	79.0	19.6	4.8	100.0
Improved water source, rural (% of rural population with access)	Water and sanitation	718	70.7	22.9	2.8	100.0
PM10, country level (micrograms per cubic meter)	Water and sanitation	724	57.0	40.2	9.8	292.1
Lag 10 years school enrollment, primary (% gross)	Education	910	94.4	25.7	14.1	207.8
Lag 10 years school enrollment, primary, female (% gross)	Education	853	89.0	28.6	1.3	180.8
Lag 5 years school enrollment, secondary (% gross)	Education	808	51.0	30.8	0.7	110.8
Lag 5 years school enrollment, secondary, female (% gross)	Education	710	48.4	32.5	0.0	111.4
School enrollment, primary (% gross)	Education	925	97.3	23.6	17.1	207.8
School enrollment, primary, female (% gross)	Education	879	92.7	25.9	1.3	180.8
School enrollment, secondary (% gross)	Education	827	56.5	30.8	2.4	110.8
School enrollment, secondary, female (% gross)	Education	736	54.9	32.6	0.0	111.4
Labor force participation rate, female (% of female population ages 15+) (modeled)	Gender equality	746	50.3	18.3	9.4	90.6
Proportion of seats held by women in national parliaments (%)	Gender equality	535	12.8	8.9	0.0	56.3
Urban population (% of total)	Urbanization	1049	45.2	21.5	4.3	94.7
Mortality rate, under-5 (per 1,000 live births)	Dependent variable	1036	79.7	67.6	3.3	331.2

^1^ N = number of country-years available for analysis in eligible time period and countries.

S.D. = standard deviation.

We examined different sources to collect data for over 250 health interventions and socioeconomic determinants of health, including the World Bank Databank, World Health Organization Global Health Observatory, and United Nations UNdata [22–24]. U5MR was the dependent variable. The “health systems” policy area included health determinants with high facility-based service delivery system needs (hospital beds, staffing coverage, antenatal care and skilled attendance at birth). Separate policy areas were created for other health determinants, including immunizations and fertility reduction, which are less dependent on 24-hour availability of facility-based service delivery systems. Fifty-two indicators with greater than 25% data availability by country-years were analyzed (Table 1) for use in multisectoral models, and 66% data availability was considered sufficient for interpretation in the univariable analysis by mortality level.

#### Time period

We used data from 1990 as our baseline year corresponding with the baseline year for the MDGs. However, we analyzed U5MR from 1980–2010 to increase statistical power.

### Statistical analysis

#### Procedures

We quantified the association between absolute changes in U5MR with absolute changes in each variable separately over five-year increments. We used five-year increments as this period is long enough to assess impact from changes in policy, but short enough for political objectives. Using five-year increments is the equivalent of using a first difference model (equations below), which controls for time-invariant unmeasured confounders, such as geography and culture. It also permits examining the effect of changes in health variables in the same country as it progresses through different mortality levels and at different times.

Data limitations included non-random missing data, outliers, unmeasured confounding from fixed effects by country, and variance by time-period across decades. To address these limitations, we employed a method previously unused for U5MR analyses, MM-estimation with first differences, and country clustering, which controls for all of these issues except non-randomness of missing data. MM-estimation is the most advanced method to control for outliers while maintaining statistical efficiency [25]. Country clustering accounts for the temporal dependence of observations from the same country. We address the issue of non-random missing data by grouping related variables within policy areas (Table 1), since each health determinant is highly correlated with many others within policy areas, but sometimes reported in different years. The regression coefficients of variables within the same policy areas therefore represent an estimate of the mortality reduction that accompanies progress in that sector.

To increase the number of data available for this regression and smooth out measurement error, variables at each five-year increment (1980, 1985, 1990, etc.) were considered to be the average of available data points in the given year, one year prior, and one year after. Since we were using first differences, imputation was not possible as first differences from imputed values were spurious.

All statistical analyses were carried out in Stata 13 [26]. A command to permit first differences, clustering by countries, and control of outliers (robust regression) was not available in Stata 13. We therefore programmed a new Stata command, xtrobdiff, which performs robust regression on first differences with MM-estimation and country clustering. The first step of this procedure, for a linear regression model of the type $y_{it}=x_{it}^{\prime }\theta +\varepsilon_{it}$, is to run a standard first difference model (Eq 1), adjusting standard errors for clustering by country *i*.

$${\hat{\theta }}_{FD}={\left(\Delta X^{\prime }\Delta X\right)}^{-1}\Delta X\prime \Delta y$$ (1)

where ***y*** is a vector of length *nT* with components *y*_*it*_ (*i* = 1,…,*n*; *t* = 1,…,*T*) and Δ***y*** is the vector of length *n*(*T* − 1) with components *y*_*it*_ − *y*_*it*−1_ (*i* = 1,…,*n*; *t* = 2,…,*T*); Δ***X*** is a matrix with *n*(*T* − 1) rows and *p* columns, whose lines are of the form (***x***_*it*_ − ***x***_*it*−1_)′; *t* = 1 represents the year 1980, *t* = 2 represents 1985, and so on.

The next step is to calculate an S-estimator ${\hat{\theta }}^{S}$, which is robust to 50% of the data being outliers.

$${\hat{\theta }}^{S}=\arg\;\min\;\sum_{l=1}^{n\left(T-1\right)}\rho_{0}\left(\frac{r_{l}\left(\theta \right)}{{\hat{\sigma }}^{S}}\right).$$ (2)

Here, *ρ*_0_(∙) represents the Tukey biweight function (with scaling parameter *k* = 1.546), a loss function which awards less importance to large residuals, and thus which optimizes the robustness of the parameter vector $\hat{\theta }$ contamination by outliers [25]. $r_{l}$ is the residual of value l. ${\hat{\sigma }}^{S}$ is the robust estimator of scale where

$$\frac{1}{n}\sum_{l=1}^{n(T-1)}\rho (\frac{r_{l}(\theta )}{{\hat{\sigma }}^{S}})=b$$ (3)

and *b* = *E*{*ρ*(*Z*)} with *Z* ∼ *N*(0,1). As detailed elsewhere [25], explicit formulas for S-estimators are not available, and it is necessary to use numerical optimization to compute them.

Finally, MM-estimation uses the S-estimator as an initial value, and then uses iteratively reweighted ordinary least squares (OLS) to increase efficiency (efficiency is the ability of the estimator to optimally test a hypothesis given a set number of observations). MM-estimation is the preferred method for robust regression, and its derivation and details have been explained elsewhere [25]. The procedure is given by Eq 4.

$${\hat{\theta }}^{MM}=\arg\min\sum_{l=1}^{n(T-1)}\rho (\frac{r_{l}(\theta )}{{\hat{\sigma }}^{S}}).$$ (4)

Here, *ρ*(∙) represents the Tukey biweight function with tuning parameter set to have the maximal acceptable efficiency [27].

We subjected each variable separately to this procedure in all four mortality groups as well as in aggregate. For each regression, we included all eligible countries in that mortality group at the baseline year of each five-year interval. Variables were considered to show a significant association with mortality decline if P < 0.05, adjusted R-squared > 0, and the sign of the coefficient, where the variable was associated with a decrease (negative sign) or increase (positive sign) in U5M, was consistent with the literature.

After analyzing each of the 52 health determinants independently, we constructed multivariable models for each mortality grouping both globally and limited to sub-Saharan Africa. We used the variable from each policy area with the strongest association with mortality reduction to build the multivariable models. Relatively high correlation between determinants meant that many combinations of variables were not possible. Because this part of the analysis was illustrative of aggregate multisectoral progress, we selected α = 0.1 as a cutoff for statistical significance. We used negative backwards elimination and iterative replacement of variables within policy areas until we found a combination of indicators that were all significant at the P < 0.1 level. The magnitude of the coefficients in the multivariable models represented the quantitative relationship between changes in each health determinant and mortality, controlling for the other variables in the model and the above statistical artifacts.

Because many of the variables varied widely in measurement unit, a common benchmark to measure the relative magnitude of the coefficients was needed. We assigned “standard improvements” for each variable using round-number five-year improvements that represented above average progress. For example, if the top quartile of countries achieved an increase in measles immunization of 12.4% over five years, the standard improvement was deemed 10%. The standard improvement was multiplied by the regression coefficient to estimate the impact of above average progress for that variable on mortality.

#### Sensitivity analysis

Using the best-fitting multivariable model, each variable was iteratively exchanged for 2 to 4 other indicators in its policy area. The coefficients from each new model were used to construct a distribution for each variable.

## Results

### Key health determinants of U5MR by baseline mortality level

Of 82 significant findings meeting the inclusion criteria of P < 0.05 and adjusted R^2^ > 0 (S2 Table), only one had a coefficient inconsistent with its hypothesized relationship to U5MR. Examination of this exception graphically (physicians per capita for total mortality) revealed it to be artefactual due to a few outliers and it was therefore excluded. This left 81 positive findings of 260 possible (31%) (Table 2). S2 Table shows the complete statistical output from the 260 regressions of the univariable analysis.

**Table 2:** Coefficients of significant findings (P < 0.05, adjusted R^2^ > 0, sign consistent with literature) for 52 variables in the univariable analysis, divided by policy area^1^.

Policy Area				U5MR				% complete
		Total	High	Moderate	Low	Very Low	N	
	Variable		(>180)	(100.1–180)	(50.1–100)	(<50)		
GDP per capita	Log GDP per capita, PPP (constant 2011 international $)	-6.454	-12.733		-15.175	-3.45	708	68%
	Log GDP per capita (constant 2005 US$)	-4.571	-18.712		-8.139		924	89%
	Debt forgiveness grants per capita (2005 US$)		-0.412			-0.014	340	33%
Poverty	GINI index						368	36%
	Poverty gap at $2 a day (PPP) (%)	0.404					370	36%
	Poverty gap at $1.25 a day (PPP) (%)	0.45					370	36%
Health systems	Births attended by skilled health staff (% of total)	-0.492				-0.278	478	46%
	Pregnant women receiving prenatal care (%)	-0.356	-0.606				340	33%
	Hospital beds (per 1,000 people)			-1.957			675	65%
	Nurses and Midwives (per 1,000 pop)					-0.336	258	25%
	Physicians (per 1,000 people)				-6.702		812	78%
Health financing	Out-of-pocket health expenditure (% of total expenditure on health)		1.00				580	56%
	Log Health expenditure per capita, PPP (constant 2005 international $)						580	56%
Immun-izations	Newborns protected against tetanus (%)	-0.082					614	59%
	Immunization, measles (% of children ages 12–23 months)	-0.212		-0.123	-0.188	-0.049	898	87%
	Immunization, DPT (% of children ages 12–23 months)	-0.249	-0.195	-0.112	-0.154	-0.066	917	89%
Malaria or HIV	Prevalence of HIV, total (% of population ages 15–49)	2.427			2.921	1.338	540	52%
	Malaria incidence, per 1,000 pop						592	57%
Fertility	Contraceptive prevalence (% of women ages 15–49)						451	44%
	Fertility rate, total (births per woman)	5.759				2.304	1057	102%
	Lag 5yr TFR				4.492	2.93	1054	102%
Nutrition	Prevalence of wasting (% of children under 5)	0.496					345	33%
	Prevalence of undernourishment (% of population)	0.309				0.143	563	54%
	Exclusive breastfeeding (% of children under 6 months)	-0.157					234	23%
	Malnutrition prevalence, weight for age (% of children under 5)	0.773		1.204			355	34%
	Malnutrition prevalence, height for age (% of children under 5)						350	34%
Govern-ance	Rule of Law: Estimate			-20.831			611	59%
	Regulatory Quality: Estimate						602	58%
	Political Stability and Absence of Violence/Terrorism: Estimate						607	59%
	Voice and Accountability: Estimate						614	59%
	Government Effectiveness: Estimate		-20.432	-13.286			602	58%
	Control of Corruption: Estimate						602	58%
Infrastr-ucture	Electric power consumption (kWh per capita)			-0.035			687	66%
	Roads, paved (% of total roads)						493	48%
	Road Density (km/km2)	6.206				3.239	535	52%
	Rail lines (total route-km)		0.47				479	46%
Water and sanitation	Improved sanitation facilities (% of population with access)	-0.607			-0.985	-0.348	710	69%
	Improved sanitation facilities, rural (% of rural population with access)				-0.672	-0.232	713	69%
	PM10, country level (micrograms per cubic meter)						724	70%
	Improved water source (% of population with access)	-0.943				-0.349	718	69%
	Improved water source, rural (% of rural population with access)	-0.64				-0.238	718	69%
Education	Lag 10yr Pri. School Female (% gross)	-0.263	-0.194	-0.169			853	82%
	School enrollment, secondary (% gross)	-0.148			-0.28	-0.073	827	80%
	School enrollment, secondary, female (% gross)	-0.129		-0.631	-0.264	-0.071	736	71%
	School enrollment, primary (% gross)	-0.187	-0.325	-0.241			925	89%
	Lag 5yr Sec. School (% gross)			-0.467			808	78%
	Lag 5yr Sec. School Female (% gross)			-0.889			710	69%
	Lag 10yr Pri. School (% gross)	-0.16		-0.169			910	88%
	School enrollment, primary, female (% gross)	-0.264	-0.326	-0.287			879	85%
Gender equality	Labor force participation rate, female (% of female population ages 15+)						746	72%
	Proportion of seats held by women in national parliaments (%)						535	52%
Urban-ization	Urban population (% of total)	-0.32			-1.13	-0.262	1049	101%

^1^N = number of country-years available for regression.

% complete = N/1036 (for the 1036 available U5MR values).

The univariable analysis had two key findings. First, some of the policy areas did not have enough data to analyze quantitatively. For example, health system strength had only one variable, physicians per capita, with greater than 66% data availability. The absence of statistically significant relationships between U5MR reduction and variables such as skilled birth attendance or prenatal care may therefore be due to missing data.

Second, for policy areas with greater than 66% data availability, some were associated with child mortality reduction across all mortality groups, while others correlated with child mortality reduction at specific mortality levels. Among the fifty-two health determinants tested, only immunization against DPT was significantly associated with child mortality reduction at all four mortality levels. If analyzed by policy area, only immunizations and education showed consistent association with child mortality reduction across all four mortality levels.

GDP per capita showed strong correlation with child mortality reduction at high, low, and very low baseline mortality, while no wealth determinant showed a correlation with child mortality for moderate baseline U5MR (between 100–180). Enrollment in primary school education was significantly correlated with mortality reduction at high and moderate mortality, and enrollment in secondary school education at moderate, low and very low mortality. Seven of eight education variables were significantly correlated with child mortality reduction at moderate baseline U5MR, suggesting a transition point in moderate mortality countries where secondary education gains in importance compared to primary education. In general, moderate mortality countries responded to a different set of effective interventions than low or very low mortality countries.

Interventions requiring higher health systems capacity such as physicians per 1,000 population were significantly associated with mortality reductions only at low mortality. Improvements in fertility, prevalence of HIV, coverage of water and sanitation (WASH), urbanization and road density also were associated with U5MR reduction where baseline U5MR was mainly low and very low mortality. Governance improvements (rule of law and government effectiveness estimates) correlated with U5MR reduction at high and moderate mortality.

### Multivariable analysis

The magnitude of association between health determinants and mortality attenuated after controlling for other variables (S2 and S3 Tables). Besides attenuation, the results from the univariable analyses were consistent with the multivariable analyses.

**Fig 1:**
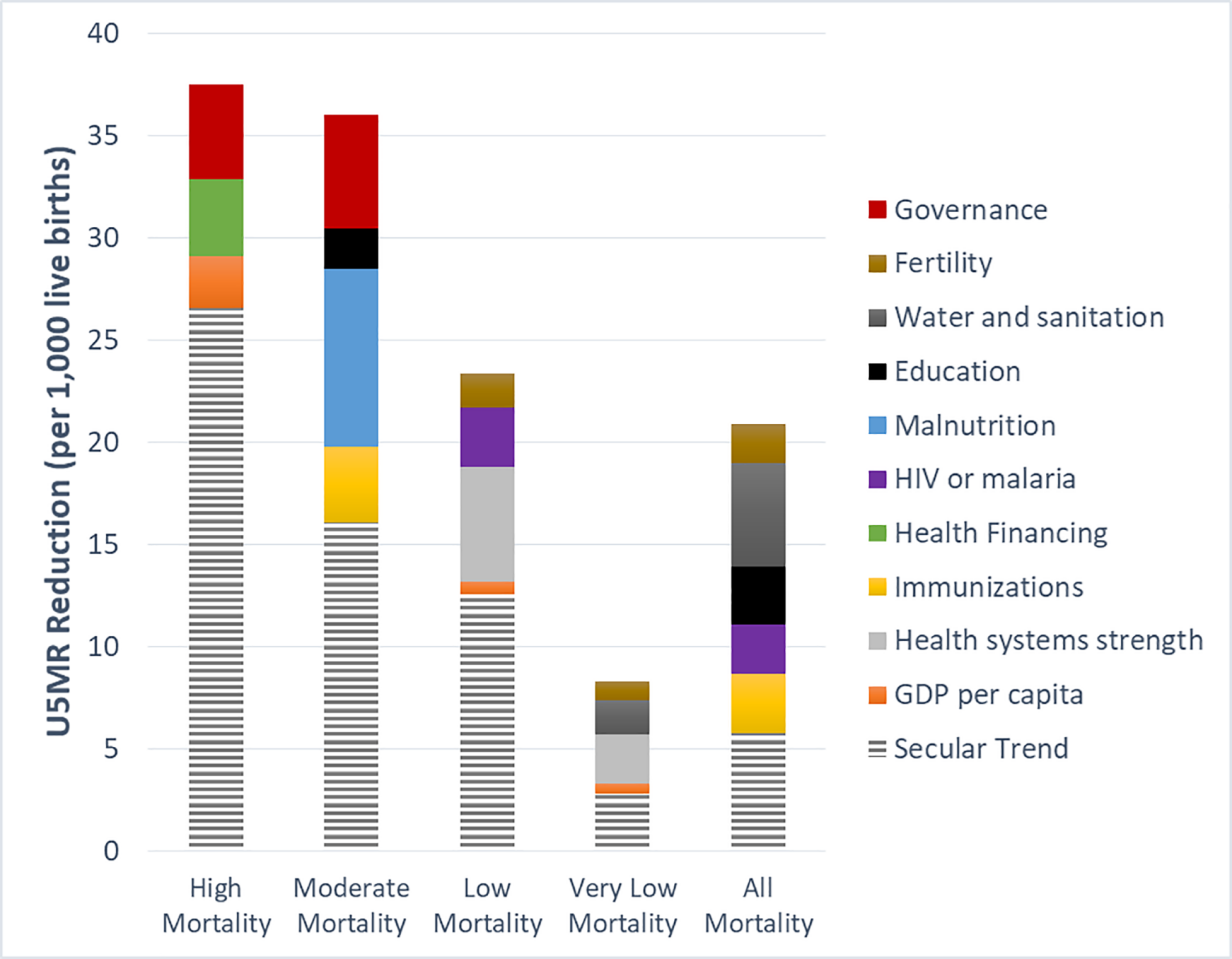
Multivariable analysis. Average five-year mortality reduction for above average multisectoral progress for U5MR, 1980–2010, by baseline mortality group. The secular trend represents the average mortality reduction in five years for zero progress in the health determinants included in the models. Additional contribution to mortality reduction from multisectoral improvements assumes standard improvements over five years in each indicator in the models as defined in the text and S4 Table.

The multivariable analysis for high U5MR was challenging given the low number of countries that comprised this mortality group. Because in 2015 no countries had U5MR > 180, this analysis focuses on the results from the moderate, low and very low groups. The secular trend contribution to the total U5MR reduction varied from 34–54% (Fig 1). The secular trend represents mortality reduction explained by anything other than the variables included in the multivariable model. In this model, improvements in four policy areas explain at least half of all mortality reduction, even given the smoothing techniques used to generate the U5MR estimates and other data quality challenges, which would inflate the secular trend. The secular trend represents the average mortality reduction in five years for zero progress in the health determinants included in the models. Additional contribution to mortality reduction from multisectoral improvements assumes standard improvements over five years in each indicator in the models as defined in the text and S4 Table.

Fig 1 shows that contributions from improvements in different health determinants combine at least additively to explain sizeable fractions of child mortality reduction. Some policy areas appear to be less contributory than others; for example, the contribution of per capita GDP improvements in the low and very low mortality groups is much smaller than the contributions from improved health system strength.

The multivariable analysis can be used to generate the expected reduction of U5MR given above average multisectoral progress (“standard improvements”) for an exemplar country of each mortality level. For example, in a moderate U5MR country, a package of standard improvements consisting of a 10% absolute increase in immunizations, a 5% absolute reduction in childhood malnutrition, a 10% increase in girls’ primary schooling, and a 0.25 increase in a country’s rule of law index lowered U5MR by 19.8 per 1000 live births over five years. This was calculated as follows: for malnutrition, the average β coefficient was β = 1.74, multiplied by a “standard improvement” 5% absolute reduction in malnutrition yields a reduction of 8.7 deaths per 1,000 live births, and so on for the other variables in the model (S4 Table). This reduction, in addition to an average secular trend reduction of 16.1 in such countries, gives a total five-year U5MR reduction of 35.9 per 1000 live births (Fig 1). The R-squared for the multivariable models was between 0.29–0.85, indicating that the 3 to 5 determinants included in the models explained 29–85% of the mortality variation (S3 Table).

Sensitivity analyses revealed a range for these estimates that were precise (S4 Table). Estimates gained in precision as data completeness increased and baseline mortality decreased. Because sub-Saharan African countries comprised over 90% of the moderate and high mortality groups, separate regressions limited to these countries were conducted. Findings for sub-Saharan Africa were comparable to those including all countries (S4K–S4N Table).

## Discussion

This paper analyzed the association between key health determinants and child mortality in LMICs across different levels of baseline mortality from 1980–2010, using existing global data.

The analysis found that multisectoral interventions were associated with 44–66% of U5MR decline across each level of country background mortality. These findings confirm the importance of multisectoral inputs to mortality reduction over time [4]. The analysis found that interventions requiring less complex public infrastructure investments or health service delivery capacity (governance, GDP, health financing, immunizations, education and nutrition), were associated with mortality decline at high or moderate mortality. Two of these policy areas–immunization coverage and education–were associated with U5MR decline across all mortality groups; and GDP at all mortality groups except moderate mortality. In contrast, some variables were associated with mortality decline only in lower mortality settings. These included HIV and malaria prevalence, fertility, water, sanitation and hygiene (WASH) improvements, human resources availability and skilled birth attendance. This finding suggests that impact of investments in these areas was not apparent until the basic building blocks of infrastructure and health systems, or social and cultural changes that come with improved education and income, or both, had been adequately developed.

The impact of education on health and mortality decline is mediated by a number of factors, both direct (better education, care practices and care-seeking) and indirect (socio-economic development, women’s employment and improvements in household wealth) [28]. The analysis suggests that these factors were operating across all mortality levels and include both primary and secondary education, with the strongest effect seen with secondary education at moderate levels of mortality. Immunization programs have relatively low facility infrastructure, equipment and supply requirements, do not require 24-hour service delivery and can be administered as part of a basic package of interventions by lower-level primary health care staff, using fixed sites, outreach from fixed sites and campaign strategies [7]. In addition, immunization programs can add new vaccines over time, thereby adding to potential effectiveness as mortality from measles and other infectious diseases declines [29]. Other primary health interventions with similar low systems requirements, deliverable using vertical programs, may also fall into this category (and measured here as secular trend). Adequate data on these interventions were not available in the global database, but could include vitamin A supplementation, malaria bed-net distribution (in endemic areas) and improved prevention, care-seeking and treatment of diarrhea and pneumonia [30, 31, 32]. Similarly, improvements in nutritional status in moderate mortality countries may be associated with both improved food security and targeted primary care programs to provide iron, de-worming and zinc supplementation and better prevent and treat diarrhea and other childhood illnesses [33].

Nutrition variables were significantly associated with mortality decline in moderate mortality settings, most strongly for declines in the proportion of children under-5 underweight (low weight for age). Declines in underweight–mediated through a number of potential mechanisms–are associated with under-5 mortality decline [34, 35]. Lack of association between mortality decline and exclusive breastfeeding may reflect relatively low national exclusive breastfeeding rates and slow gains in coverage over time [36]. The impact of nutritional improvements may be expected to be less significant in lower mortality settings where overall nutritional status has improved and where acute malnutrition and under-nutrition are less prevalent.

Variables associated with improvements in wealth influence mortality through multiple possible channels including community and household mediated effects such as improved living conditions, access to medical care, female education and other factors [8]. The association of health-care financing with mortality declines in the high mortality group suggests that these investments are mediated by primary care programs delivering basic packages of care, rather than investments in infrastructure and systems to support improved quality of clinical practice. Governance was associated with mortality decline at high and moderate mortality levels. This indicator is a composite measure of a number of aspects of government functioning, including quality of public services, the civil service, policy development and implementation [22]. It is difficult to quantify which components were most strongly associated with mortality reduction, and factors are likely to vary depending on the setting. The Success Factor studies in high performing countries suggested that establishing clear plans, policies and guidelines was essential to supporting program implementation [5].

Variables associated with mortality decline in lower mortality settings tended to have higher infrastructure and health system capacity requirements in addition to requiring socioeconomic or cultural changes for effectiveness. WASH improvements are resource intensive and require both large infrastructure investments as well behavioral changes to use these resources adequately. Coverage and widespread impact on childhood nutrition, diarrhea morbidity and chronic enteric disease is likely to require time, particularly for use of improved sanitation sources [37, 38, 39, 40]. The relationship between fertility and child mortality declines is complex. Fertility declines are hypothesized to reduce child mortality through a number of mechanisms, including prolonged birth interval, increased likelihood of exclusive and prolonged breastfeeding, and reduced births to younger and older mothers. In addition, fertility is influenced strongly by economic, educational and cultural factors that impact use of contraception and parental decisions about family size–and in turn are associated with mortality risk [41,42]. This analysis found that that measurable associations between fertility and mortality declines were not seen until lower mortality settings. Similarly, declines in national prevalence of HIV and malaria require changes in many environmental factors, some of which are related to improved prevention and public infrastructure; and all requiring behavioral change. The prevention of mother to child transmission of HIV requires better access to and quality of antenatal, delivery and postpartum care that is has proved challenging in LMICs and requires both improved staff availability, skills and resources for testing and treatment [43, 44]. Improved quality of maternal, newborn and child care has been difficult to achieve even when access to care improves [45, 46, 47]. This analysis confirms that these more complex improvements in quality have not been historically associated with significant mortality reductions until mortality was lower, suggesting that investments in these areas must be made well before impact on mortality is quantifiable.

How can policy makers interpret these findings? First, investments in the health and non-health sectors were required to maximally reduce U5MR, so all fourteen policy areas included in this analysis should be considered and lagging areas identified. These investments build on and complement each other. Second, the association of some interventions with mortality decline varies with baseline mortality, level of health systems functioning and socioeconomic progress. Achieving adequate coverage and quality for some interventions required greater investments over time than others before national mortality impact was quantifiable. Third, significant mortality reduction can be achieved, despite suboptimal GDP, health financing and governance. These gains are associated with the adoption of multisectoral approaches, mobilization of partners, and accountable stakeholder actions in resource-poor settings [21]. In summary, these data can aid planning for where to allocate resources and for calculating realistic targets based on real historical progress among countries with similar mortality profiles.

Cross-country analyses of associations between mortality decline and health determinant improvements present several challenges. Although there are many indicators publicly available, data are often incomplete, especially among the highest mortality countries [48]. Indicators for determinants of greatest interest, such as health system strength, are either unavailable or available only as proxy measures for other un-measured interventions. Data may be of varying quality, the product of models, have outliers, or only available over a short time period, reducing the reliability in any model. Lucas showed that cross-country regressions can be sensitive to the sample of countries included in a particular regression [49]. Socioeconomic determinants of health are often highly correlated, making it difficult to include many in the same regression model. The relatively large size of the secular trend, which has been observed in similar analyses [50], may result from smoothing of the outcome variable or with problems building models containing all the true drivers of mortality decline.

This analysis accounted for most of these issues, of which non-random missing data and outliers were most important. In the univariable analysis, 29% of regressions yielded statistically significant results, well above what would be suggested by chance. MM-estimation is the most advanced method to control for outliers while maintaining statistical efficiency [25], and we combined this with other necessary control measures for time series data, namely first differences and country clustering. To account for the problem of variably missing data, we grouped indicators into policy areas since many indicators within policy areas were correlated. We focused on how different sectors contributed in an additive way to mortality decline. We additionally conducted sensitivity analyses that showed a stable range for coefficients within policy areas from many different regressions with many patterns of missing data.

Our analysis differs in approach from the Lives Saved Tool (LiST) [51]. We quantify the effect of historical data as it actually transpired, controlling for statistical issues such as fixed country effects, secular trends over time, unmeasured confounding, and outliers. We also emphasize overall impact from multisectoral improvements. LiST generates future projections using meta-analysis-derived quantitative estimates of the effectiveness for multiple separate interventions on cause-specific mortality given a country’s starting population structure, disease burden, and intervention coverage, and therefore is based on well-validated but theoretical predictions which also do not model secular time trends. Both approaches have merit, and it would be interesting to compare plans produced by the two as well as their results.

## Conclusions

As countries plan to achieve the Sustainable Development Goals, they will utilize evidence-based interventions from many policy areas. For higher mortality countries, some of the global SDG targets, such as U5MR of 25 per 1,000 live births by 2030, will require considerable additional resources and multisectoral progress to be achieved [29]. Country-specific targets and programs that take into account the current mortality level are needed [33]. Analyses such as this can quantify the likely mortality reduction after implementation of an ambitious but achievable plan of multisectoral improvements.

## Supporting information

S1 Table Countries included in the analysis. Countries are listed by region and number times they counted under each mortality grouping in five-year increments from 1980–2010.
(XLSX)

S2 Table Under-5 mortality univariable regression results. Full regression output for each univariable regression analysis by variable and mortality grouping.
(XLSX)

S3 Table Multivariable regression results. Regression results for each mortality group.
(XLSX)

S4 Table Sensitivity analyses results. Results from sensitivity analysis including range of coefficients by policy area and impact on under-5 mortality rate under conditions of standard improvements (as defined in Methods) in each of the policy areas included in the multivariable regression model.
(XLSX)

S5 Table Cohen_etal_FinalData.xlsx. Full dataset used in the analysis.
(XLSX)
